# Isoform Specific Effects of Mef2C during Direct Cardiac Reprogramming

**DOI:** 10.3390/cells9020268

**Published:** 2020-01-22

**Authors:** Li Wang, Peisen Huang, David Near, Karan Ravi, Yangxi Xu, Jiandong Liu, Li Qian

**Affiliations:** 1 McAllister Heart Institute, University of North Carolina at Chapel Hill, Chapel Hill, NC 27599, USA; wali@email.unc.edu (L.W.); hps0814@sina.com (P.H.); dave97@live.unc.edu (D.N.); karan97@live.unc.edu (K.R.); yangxixu@unc.edu (Y.X.); jiandong_liu@med.unc.edu (J.L.); 2 Department of Pathology and Laboratory Medicine, University of North Carolina at Chapel Hill, Chapel Hill, NC 27599, USA; 3 Department of Cardiology, The First Affiliated Hospital of Sun Yat-Sen University, Guangzhou 510080, China; 4 NHC Key Laboratory of Assisted Circulation (Sun Yat-sen University), Guangzhou 510080, China

**Keywords:** fibroblasts, Mef2C protein, isoform, cardiac myocytes, reprogramming

## Abstract

Direct conversion of cardiac fibroblasts into induced cardiomyocytes (iCMs) by forced expression of defined factors holds great potential for regenerative medicine by offering an alternative strategy for treatment of heart disease. Successful iCM conversion can be achieved by minimally using three transcription factors, Mef2c (M), Gata4(G), and Tbx5 (T). Despite increasing interest in iCM mechanistic studies using MGT(polycistronic construct with optimal expression of M,G and T), the reprogramming efficiency varies among different laboratories. Two main Mef2c isoforms (isoform2, Mi2 and isoform4, Mi4) are present in heart and are used separately by different labs, for iCM reprogramming. It is currently unknown if differently spliced isoform of Mef2c contributes to varied reprogramming efficiency. Here, we used Mi2 and Mi4 together with Gata4 and Tbx5 in separate vectors or polycistronic vector, to convert fibroblasts to iCMs. We found that Mi2 can induce higher reprogramming efficiency than Mi4 in MEFs. Addition of Hand2 to MGT retroviral cocktail or polycistronic Mi2-GT retroviruses further enhanced the iCM conversion. Overall, this study demonstrated the isoform specific effects of Mef2c, during iCM reprogramming, clarified some discrepancy about varied efficiency among labs and might lead to future research into the role of alternative splicing and the consequent variants in cell fate determination.

## 1. Introduction

Ischemic heart disease is the leading cause of mortality worldwide, accounting for 8.93 million deaths per year [1]. After myocardial infarction (MI), massive and permanent loss of cardiomyocytes (CMs) leads to cardiac malfunctions and eventually heart failure. Developing new strategies to replenish lost CMs to restore heart function remains a big challenge to be addressed.

Recent advances in direct cardiac reprogramming provide a promising approach by converting fibroblast into induced cardiomyocytes (iCMs), for regenerating damaged heart tissue in situ. Ectopic expression of cardiac-lineage transcription factors (TFs) could directly reprogram fibroblasts into iCMs, without going through the pluripotent stage [2,3,4,5,6,7]. Delivery of minimal three TFs Mef2c (M), Gata4 (G), and Tbx5 (T) has been reported to successfully generate murine iCMs from fibroblasts, both in vitro and in vivo [6,7]. Our previous work demonstrated that polycistronic expression of the optimal ratio of M,G,T factors further improved reprogramming efficiency and enhanced heart function recovery, in a murine MI model [8,9]. Meanwhile, increasing efforts have been focused on developing new strategies to further increase iCM reprogramming efficiency, yield and purity, including addition of extra TFs [5,10,11], microRNAs [2] and chemicals [12,13,14,15,16], genetic and epigenetic manipulations [17,18], and adoption of different viral delivery platforms [19]. However, the base reprogramming efficiency varies among different laboratories, which hinders the reproducibility of mechanistic studies and further translational applications.

Mef2c is one of the master regulators governing cardiac gene regulatory network and heart development. During direct cardiac reprogramming, efficient iCM conversion requires relatively high protein level of Mef2c [9]. Enhanced transcriptional activity of Mef2c to the promoter regions of cardiac structural genes, remarkably promotes iCM reprogramming [20]. Till date, iCM researchers have mainly used two differently spliced isoforms of Mef2c. It remains elusive whether different Mef2c isoforms contribute to different outcome of reprogramming events.

In this study, we revealed the distinct expression of two major Mef2c isoforms (Mef2c isoform 2, Mi2 and Mef2c isoform 4, and Mi4) in murine primary cardiomyocytes and different types of fibroblasts. Furthermore, we demonstrated that polycistronic Mi2-Gata4-Tbx5 (Mi2GT) is more efficient than Mi4-Gata4-Tbx5 (Mi4GT) in generating iCMs from mouse embryonic cells (MEFs), but not from cardiac fibroblasts (CFs) or tail-tip fibroblasts (TTFs). Addition of Hand2 enhanced iCM conversion using separate G, T, and M (either Mi2 or Mi4) factors and Mi2GT polycistronic construct. These findings provided the first piece of evidence showing Mef2c splicing variants do constitute one of the major contributors to seemingly inconsistent reprogramming efficiencies among different laboratories. In addition, the results highlighted the importance of isoform specific transcriptional regulation during cellular reprogramming that might be worth future investigation beyond the iCM induction process.

## 2. Materials and Methods

### 2.1. Plasmids

Retroviral plasmid encoding Mef2C isoform 4 (Mi4) was a gift from Kunhua Song (University of Colorado School of Medicine) [5]. Retroviral vectors encoding mouse Gata4, Mef2C isoform 2 (Mi2), and Tbx5 in pMXs based vectors were described previously [7,21]. The polycistronic constructs of Mi2GT and Mi4GT were generated with the protocol described previously [9].

### 2.2. Viral Packaging and Transduction

Plat-E packaging cells were maintained in culture media containing DMEM plus 10% FBS, 50 units/50 μg/mL penicillin/streptomycin, 1 μg/mL puromycin (Sigma, St. Louis, MO, USA), and 100 μg/mL of blasticidin S (Life Technologies, Carlsbad, CA, USA). One day before transfection, 6 × 10^6^ cells were seeded onto a 10-cm dish, in culture media, without puromycin and blasticidin. The next day, pMXs-based retroviral vectors were introduced into Plat-E cells using Lipofectamine 2000 transfection reagent (Life Technologies), according to the manufacturer’s recommendations. Generally, 10 μg of plasmid DNA, diluted with a 500-μL Opti-MEM media (Life Technologies) was added to another 500-μL Opti-MEM media containing 20 μL of Lipofectamine, after 5 min of incubation. The mixture was incubated at room temperature for 20 min before being added to the Plat-E cells. Cells were then incubated overnight at 37 °C with 5% CO_2_. The medium was changed the next day and the virus containing-supernatant was collected 48 h after transfection, followed by filtration through a 0.45 μm filter (Thermo Scientific, Waltham, MA, USA) and incubation with a Retrovirus Precipitation Solution (ALSTEM, CA, USA), overnight. Viruses were then re-suspended by a fibroblast media supplemented with 4 μg/mL polybrene (Life Technologies) and were immediately added to the target cells. Forty-eight hours after infection, the virus containing medium was replaced with an iCM medium (10% FBS of DMEM/M199 (4:1)) and changed every 2–3 days. For positive selection, puromycin at 2 μg/mL was added to the cells three days after viral infection and was maintained in an iCM medium, at a concentration of 1 μg/mL.

### 2.3. Isolation of MEFs

Embryos from αMHC-GFP reporter mice at E14.5 were harvested and their internal organs and head were removed, as previously described [18]. The body below the head was minced to small pieces. Minced embryos were incubated with 1 mL of 0.05% trypsin/1mM EDTA (Gibco) and 100 U/mL DNase for 15 min at 37 °C with 5% CO_2_. Cells were suspended in 25 mL of cultural medium (DMEM/10% FBS, 50 units/50 μg/mL penicillin/streptomycin, and 1% GlutaMAX supplement (Gibco)) and then plated on a 10-cm dish. In 24 h, the media were aspirated, and a new 10 mL of growth medium was added. In 72 h, MEFs were harvested and stored for future use.

### 2.4. Derivation of Neonatal Fibroblasts

For enzyme digestion, hearts were removed from αMHC-GFP transgenic mice and rinsed thoroughly with chilled PBS to remove blood and other tissues. The hearts were then minced roughly into 1 mm × 1 mm pieces, transferred to 20 mL warm 0.05% Trypsin-EDTA, and incubated at 37 °C for 20 min. The supernatant was discarded, and the heart pieces were digested with 10 mL warm 0.2% collagenase type Ⅱ in HBSS (Hanks’ Balanced Salt solution), for 7 min at 37 °C, followed by vortexing for 1 min. The supernatant was collected and diluted with 7 mL fibroblast medium. After five rounds of collagenase digestion and collection, single cell suspension was obtained by passing through 40 μm cell strainer. Cell pellet was surrendered in 1 mL of red cell lysis buffer (150 mM NH_4_Cl, 10 mM KHCO_3_, and 0.1 mM EDTA), for one minute on ice, and was resuspended in an MACS (magnetic cell sorting) buffer (DPBS (Dulbecco’s Phosphate Buffered Saline) with 0.5% BSA and 2 mM EDTA (Ethylenediaminetetraacetic acid)) for sorting. For the explant culture, isolated neonatal (day 1–3) hearts or tail tips from αMHC-GFP transgenic mice were minced into 1 mm × 1 mm pieces and placed onto 10-cm dishes with 2 mL explant medium (IMDM/20% FBS). Three hours later, when the tissue pieces settled down, another 10 mL medium was slowly added and was replaced every three days. Migrated cells at day 7 were harvested and filtered through 40 μm cell strainers for cell sorting.

### 2.5. Magnetic Cell Sorting

Thy1.2 positive fibroblast cells, obtained from either enzyme digestion or explant culture, were isolated by Magnetic-activated cell sorting (Miltenyi Biotec, Bergisch Gladbach, Germany), according to the manufacturers’ instructions. Briefly, cells (about 1 × 10^7^) were suspended in 90-μL MACS buffer and incubated with 10-μL Thy1.2 Biotin anti-Mouse CD90.2 antibody (eBioscience, Inveitrogen, San Diego, CA, USA), for 30 min. After one MACS buffer wash, the cells were suspended in another 90-μL MACS buffer and incubated with 10-μL Anti-Biotin MicroBeads (Miltenyi Biotec), in a refrigerator, for 30 min. After two MACS buffer washes, the cells were passed through a 30-μm nylon mesh and applied to the calibrated LS column. The target cells were flushed out after two washes and were resuspended in explant culture medium for further usage.

### 2.6. Flow Cytometry

Adherent cells were washed with PBS. Cells were detached from the culture dish by treatment with 0.05% Trypsin/EDTA, for 5 min at 37 °C. Cells in trypsin were neutralized with 1 mL of 4% FBS/PBS and fixed with 0.2 mL of BD Cytofix/Cytoperm solution, for 30 min on ice. Cells were washed with 500 μL of BD Perm/wash buffer twice and then incubated with 50 μL of primary antibody against mouse Troponin T (cTnT, Thermo Scientific, 1:400) and rabbit GFP (Invitrogen, 1:500) in BD Perm/Wash buffer, for 30 min at 4 °C. Cells were washed with 500 μL of cold BD Perm/wash buffer and incubated with the secondary antibody, Alexa Fluor 647-conjugated donkey anti-mouse IgG, and Alexa Fluor 488-conjugated donkey anti-rabbit IgG (Jackson ImmunoResearch Inc., 1:500) in 50 μL BD Perm/wash buffer, for 30 min at room temperature (RT). Cells were washed with 1 mL cold BD Perm/wash buffer and re-suspended in 200 μL of 1% paraformaldehyde/PBS and then analyzed using the Cyan (Beckman Coulter) and FlowJo software (Treestar).

### 2.7. Immunocytochemistry (ICC)

Cells were washed with ice cold PBS for three times and fixed with 4% paraformaldehyde at RT for 15 min and then washed with PBS for three times. After permeabilization with 0.1% Triton X-100/PBS for 20 min and blocking in 5% BSA for 1 h, cells were treated with primary antibody at 4 °C, overnight. Cells were washed with PBS three times and then incubated with secondary antibody for 1 h at RT, and subsequent nuclei staining with Hoechst (Molecular Probes 33342, 1:5000), for 1 min at RT. The following primary antibodies were used—cardiac troponin T (Thermo Scientific, 1:400), GFP (Invitrogen, 1:500), α-actinin (Sigma-Aldrich, 1:500), Connexin43 (Sigma-Aldrich, 1:200), and Mef2C (Abcam, 1:1000). Images were acquired using EVOS^®^ FL Auto Cell Imaging System (Life Technologies).

### 2.8. Quantitative Real-Time qPCR

RNA was extracted with Trizol (Invitrogen, Carlsbad, CA, USA). Frist strand cDNAs were synthesized by using the Superscript IV first-strand synthesis system (Invitrogen). qRT-PCR was performed using the ABI ViiA 7 Real-Time PCR system (Applied Biosystems, Foster City, CA, USA) and the Power SYBR Green PCR Master Mix (Applied Biosystems), according to the manufacturer’s protocols. mRNA levels were normalized to those of *Gapdh*.

### 2.9. Western Blots

Cells were collected and lysed in 4 × SDS loading buffer (Bio-Rad) and subjected to SDS-PAGE. After separation, proteins were transferred to nitrocellulose membranes and probed with the indicated antibodies. The target proteins were detected by chemiluminescence (ECL, Thermo Scientific). The membranes were stripped with stripping buffer (Sigma) and re-probed with antibody against a second protein or β-Actin for loading control.

### 2.10. Chromatin Immunoprecipitation (ChIP) Followed with qPCR (ChIP-qPCR)

ChIP was performed with MEFs at post-transduction day 3 according to a previously described protocol, with the following optimizations [18,22]. Briefly, 5–10 million cells were cross-linked using 1% paraformaldehyde/PBS at room temperature for 10 min and were sheared using Bioruptor (30s on/60s off × 14 cycles). The sheared chromatin DNA–protein complex was then precipitated with anti-Histone H3 (acetyl K27) antibody (Abcam (ab4729), 5 µg per ChIP) and Protein G Dynabeads™ (Thermo), at 4 °C overnight. The immunoprecipitated products were washed sequentially, as previously described [18] and eluted in elution buffer (1% SDS, 0.1 M NaHCO3) at 68 °C, with a 1,400 rpm agitation for 40 min. Decrosslinking was performed by incubation in 42 °C for 2 h and then 67 °C for 6 h. The DNA was further purified by QIAquick PCR Purification Kit (Qiagen, Hilden, Germany) and then used for qPCR, using the indicated primers as described previously [22].

### 2.11. Statistical Analysis

Data were given as mean ± standard error of mean (SEM). Continuous variables were compared by the Student *t*-test. Comparison of multiple groups was performed by one-way ANOVA. Data were analyzed with SPSS 22 (IBM) or GraphPad Prism 7 for Windows, version 7.01 (GraphPad Software, Inc.). Graphs were assembled in GraphPad Prism 7. *p* < 0.05 was considered to indicate significant difference.

## 3. Results

### 3.1. Expression of Mef2C Isoforms in Primary Cardiomyocytes and Fibroblasts

**Figure 1:**
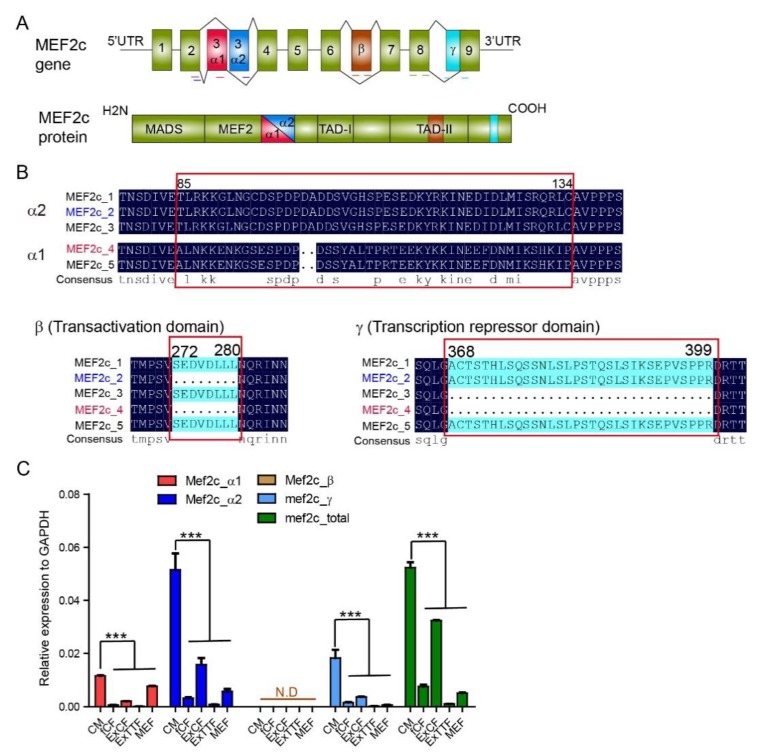
Expression of Mef2C isoforms in primary cardiomyocytes and fibroblasts. (**A**) Schematic illustration of Mef2C isoforms and location of specific primers. (**B**) Protein sequence alignment of α, β, and γ domain in different Mef2C isoforms. Differentially expressed sequences are marked in red frame and similar and identical residues are marked with dots. (**C**) qPCR analysis of Mef2C isoforms expression in cardiomyocytes (CM), fresh cardiac fibroblasts (fCFs), explanted cardiac fibroblasts (ExCFs), explanted tail tip fibroblasts (ExTTFs), and mouse embryonic fibroblasts (MEFs). For each experiment, n = 3; averaged numbers from technical triplicates were used for statistics. All data are mean ± SEM. *** *p* < 0.001.

Murine Mef2c gene consists of 9 exons with a variably included β region between exon 6 and exon 7, coding for multiple splice variants that share a conserved N-terminal MADS (MCM1-agamous-deficiens-serum response factor) box and an MEF (myocyte-specific enhancer factor) domain (Figure 1A) [23,24]. These domains are essential for DNA binding and for interaction with myogenic basic helix–loop–helix proteins (bHLH) [25]. The third exon in Mef2c variants is either exon 3α1 or exon 3α2, spliced in a mutually exclusive manner. Around 40% of nucleotide sequences are conserved between Mef2c α1 and α2 extron. The α2-Mef2c variant has been reported to be primarily expressed in the skeletal muscle, whereas the α1-Mef2c variant is expressed in other tissues [26]. The β exon encodes for the second transcription activation domain (TAD) and is expressed in neuronal tissues, including the brain [26,27]. The γ exon included in some Mef2c variants showed strong transcription repressive function. We performed sequence alignment of five main Mef2c variants and found that the commonly used Mef2c for direct reprogramming has two distinct isoforms. One variant (MEF2c_2, short for Mi2 hereafter) that contains α2 extron and γ exon and another (MEF2c_4, short for Mi4 hereafter) that contains only α1 extron (Figure 1B).

We next evaluated the expression of Mef2c variants by qPCR analysis, using exon-specific primers (shown in Figure 1A) in murine primary cardiomyocytes (CMs) and different types of fibroblasts, including freshly isolated cardiac fibroblasts (fCFs), explanted cardiac fibroblasts (ExCFs), explanted tail tip fibroblasts (ExTTFs), and mouse embryonic fibroblasts (MEFs). Primers targeting universal exon 8 were used to detect the overall expression of Mef2c. The expression level of Mef2c variants was the highest in CMs than in other fibroblasts. While β exon expression was barely detected, expression of Mef2c variants with α1, α2, and γ exons exhibited similar expression patterns among the examined cell types. Cardiac fibroblast exhibited an elevated level of Mef2c variants than tail tip fibroblast. Importantly, Mef2c variant with α2 exon was highly enriched in primary CMs than in other exons (Figure 1C).

### 3.2. Mef2C Isoform 2 Induced Higher iCM Reprogramming Efficiency in MEFs When Using Polycistronic Construct

**Figure 2:**
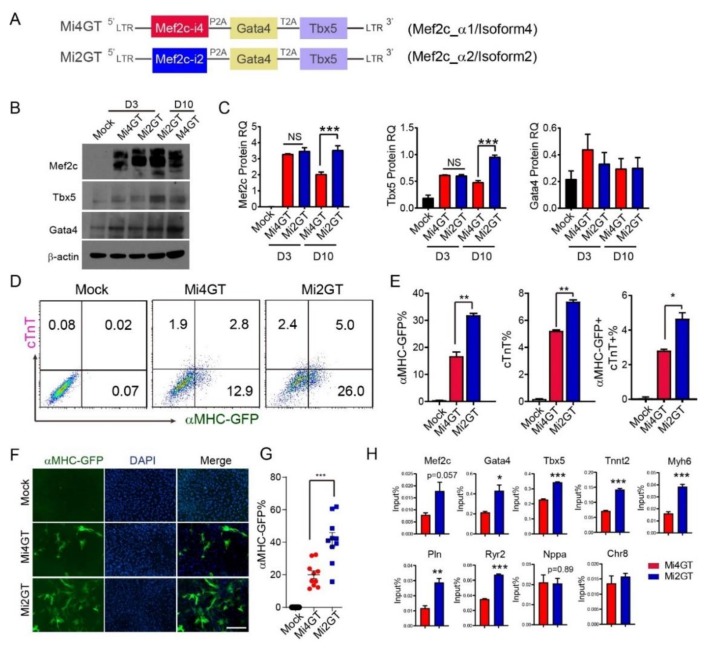
Mef2C isoform2 enhances iCM reprogramming in mouse embryonic cells (MEFs). (**A**) Schematic illustration of polycistronic constructs of Mi4GT and Mi2GT. (**B**,**C**) Western blot (**B**) and quantification (**C**) of M/G/T expression from Mi4GT and Mi2GT retrovirus infected MEF on day 3 and day 10. (**D**) Flow cytometry analysis and quantification (**E**) of iCMs generated from Mi4GT and Mi2GT transduced MEFs. (**F**) ICC analysis and (**G**) quantification of iCMs generated from Mi4GT and Mi2GT transduced MEFs. Green color represents αMHC-GFP+ cells. (**H**) ChIP-qPCR for H3K27me3 using iCMs transduced with Mi4GT or Mi2GT retrovirus on reprogramming day 3. Enrichment of indicated cardiac marker genes were evaluated and normalized to the value of input, respectively. Amplification of an intragenic region in chromatin 8 (Chr8) was used as a negative control for ChIP-qPCR. For each experiment, n = 3–4; the averaged numbers from technical triplicates, or at least 10 random ICC fields were used for statistics. All data are mean ± SEM. Statistical analysis was performed with one-way ANOVA, followed by Tukey’s test. * *p* < 0.05, ** *p* < 0.01, *** *p* < 0.001.

To identify the biological function of Mef2c variants during cardiac reprogramming, we first generated two polycistronic constructs to include Mef2c isoform 2 (Mi2, with α2 exon) or Mef2c isoform 4 (Mi4, with α1 exon) with Gata4 (G), and Tbx5 (T), in a single mRNA, as previously described [9] (Figure 2A). We termed the two constructs as Mi2GT and Mi4GT, respectively. To evaluate the relative levels of G, M, and T protein expression, we transduced MEFs with Mi2GT and Mi4GT constructs separately. Western blot analysis showed that G, M, or T proteins were overexpressed at the appropriate molecular weight and at a similar ratio compared to the loading control. On reprogramming day 3, Mef2c and Tbx5 expression was similar in MEFs transduced with either Mi4GT or Mi2GT. In comparison, on reprogramming day 10, both Mef2c and Tbx5 expression was dramatically higher in Mi2GT transduced cells than that in Mi4GT-cells (Figure 2B,C). Gata4 protein level was indistinguishable between the two constructs, at both day 3 and day 10 (Figure 2B,C).

We next sought to determine iCM reprogramming efficiency using Mi2GT and Mi4GT polycistronic constructs, respectively. We isolated MEFs from αMHC-GFP reporter mice as, described previously [7,28]. Activation of the GFP reporter allowed us to follow the emergence of newly induced iCMs. Furthermore, we used cardiac Troponin T (cTnT) as an additional CM marker to monitor the CM fate induction. We transduced MEFs with retrovirus encoding Mi2GT or Mi4GT polycistronic constructs. Both flow analysis and immunocytochemistry (ICC) quantification showed that Mi2GT delivery resulted in significantly enhanced reprogramming efficiency, compared with the Mi4GT vector (Figure 2D–G). These results revealed that the Mi2 variant enhanced iCM reprogramming in MEFs, compared to the Mi4 variant in polycistronic constructs.

Our previous studies have demonstrated the early repatterning of epigenetic landscapes during iCM fate conversion [22]. We next sought to explore if Mef2c isoform affects active epigenetic modifications of iCMs. We, thus, evaluated the enrichment of acetyl Histone H3 at the promoter region of cardiac genes by ChIP-qPCR in Mi2GT and Mi4GT-transduced iCMs, on reprogramming day 3. Interestingly, transduction of Mi2GT significantly increased acetyl H3K27 of the tested promotor, including Gata4, Tbx5, and other cardiac genes, indicating the more active chromatin states in Mi2GT-induced iCMs (Figure 2H).

### 3.3. Mef2C Isoforms Yielded Similar Reprogramming Efficiency in CFs and TTFs Using Polycistronic Constructs

**Figure 3:**
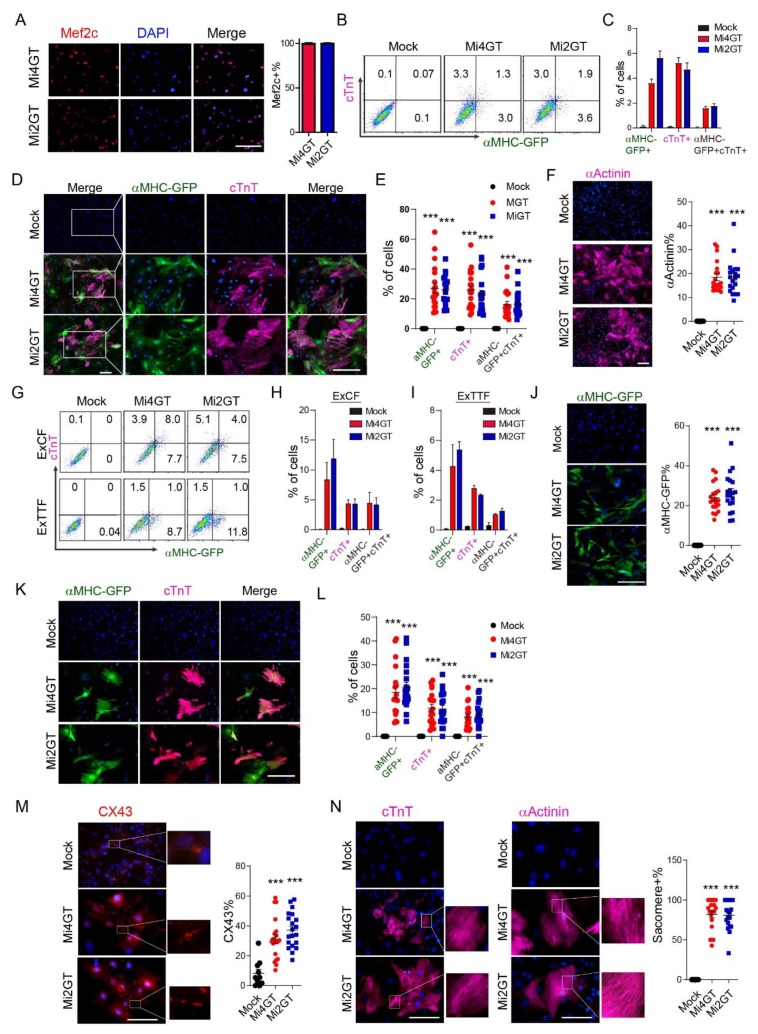
Mef2C isoforms yielded similar reprogramming efficiency in cardiac fibroblasts (CFs) and tail-tip fibroblasts (TTFs). (**A**) Immunofluorescence analysis of Mef2C expression in Mi4GT and Mi2GT retrovirus infected cells. (**B**) Representative flow cytometry plots of cTnT+, αMHC-GFP+, and double positive iCMs generated from fCF. (**C**) Quantification of the flow cytometry data. (**D**) Representative immunocytochemistry (ICC) images of iCMs generated using polycistronic constructs, Mi4GT and Mi2GT. (**E**) Statistical analysis of the immunocytochemistry data. (**F**) Representative ICC images and quantification of αActinin expression in the generated iCM from fCFs. (**G**) Representative flow cytometry plots of cTnT+, αMHC-GFP+, and double positive iCMs generated from ExCF and ExTTF. Quantification of the flow cytometry data of ExCF (**H**) and ExTTF (**I**). (**J**–**L**) Representative ICC images and quantification of the αMHC-GFP+ and cTnT+ cells, in iCMs generated from ExCF and ExTTF, using Mi4GT and Mi2GT. (**M**) Representative ICC images of connexin 43 (CX43) between both iCMs generated by Mi2GT and Mi4GT. (**N**) Representative ICC images of the sarcomere structure in iCMs generated by Mi2GT and Mi4GT. Scale bar, 200 μm. For each experiment, n = 3; averaged numbers from technical triplicates, or at least 10 random ICC fields were used for statistics. All data are mean ± SEM. *** *p* < 0.001. Statistical analysis was performed with one-way ANOVA, followed by Tukey’s test.

We next assessed the relative efficiency of Mi2 and Mi4 for MGT((polycistronic construct with optimal expression of M, G and T))-mediated reprogramming in freshly isolated neonatal cardiac fibroblasts. Nuclear expression of Mef2c was similar between Mi2GT and Mi4GT transduced cells on reprogramming day 14 (Figure 3A). Delivery of both constructs in fCFs led to generation of αMHC-GFP+ or cTnT+ iCMs. Flow analyses demonstrated no statistically significant differences in terms of percentage of αMHC-GFP+ or cTnT+ cells in both Mi2GT- and Mi4GT-infected cells (Figure 3B,C). Quantification of ICC analyses also revealed that a similar level of αMHC-GFP+, cTnT+ and αActinin+ cells were generated by using Mi2GT and Mi4GT (Figure 3D–F).

We next used other cell origins of fibroblast, including neonatal explant cardiac fibroblasts (ExCFs) and tail tip fibroblasts (ExTTFs). Upon transduction of Mi2GT and Mi4GT, both ExCFs and ExTTFs exhibited marginal differences in the percentage and absolute number of cardiac-marker-positive iCMs, using both flow and ICC analyses (Figure 3G–L). To further validate this result, we performed immunostaining for connexin 43 (Cx43, also known as GJA1), the major connexin in functional cardiomyocyte [29]. Gap junctions were observed between both iCMs generated by Mi2GT and Mi4GT, but the percentage of coupling of reprogrammed iCMs showed minimal difference (Figure 3M). We also assessed the expression of αActinin and found that these two Mef2c isoforms produced similar levels of the sarcomere structure (Figure 3N). Taken together, these results suggested that two Mef2C isoforms induced similar reprogramming process in CFs and TTFs, when polycistronic constructs were used.

### 3.4. Addition of Hand2 Enhanced Both Mef2C Isoforms Mediated iCM Reprogramming when Using Separate Factors

**Figure 4:**
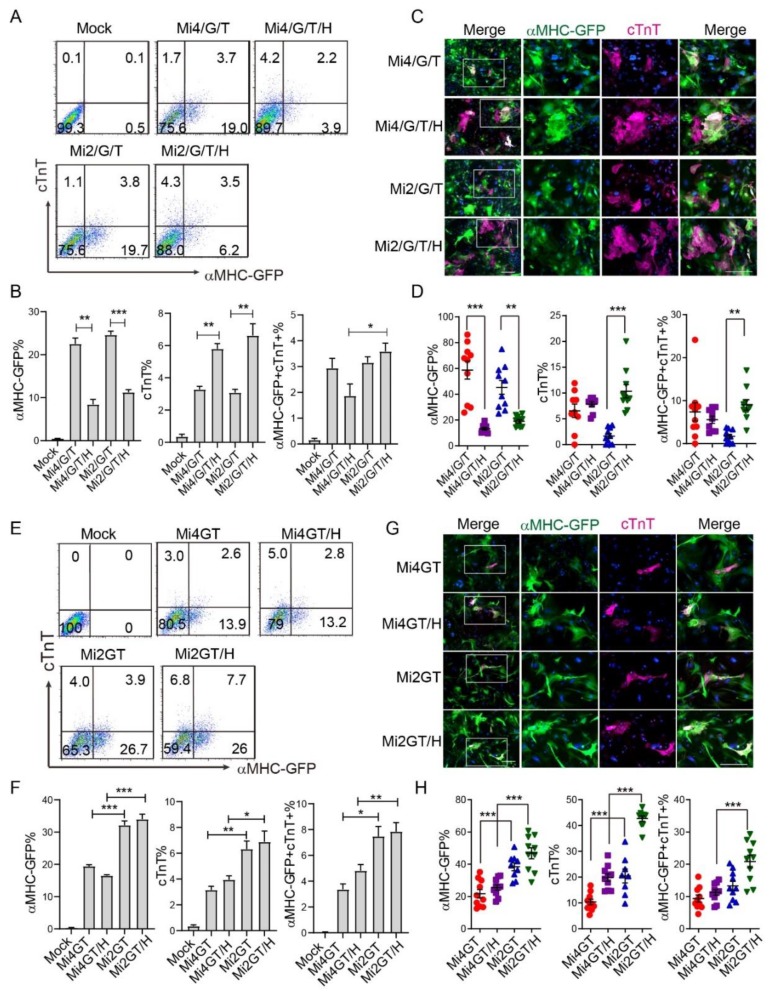
Role of Hand2 in different Mef2C isoforms mediated iCM reprogramming. (**A**) Representative flow cytometry plots of cTnT+, αMHC-GFP+, and double positive iCMs generated from MEF using Mi2/G/T or Mi4/G/T separate vectors plus Hand2. (**B**) Quantification of the flow cytometry data. (**C**) Representative ICC images of iCMs generated using M/G/T separate vectors plus Hand2. (**D**) Statistical analysis of the ICC data in C. (**E**) Representative flow cytometry plots of cTnT+, αMHC-GFP+, and double positive iCMs generated from MEF using polycistronic Mi2GT or Mi4GT constructs plus Hand2 vector. (**F**) Quantification of the flow cytometry data in (**E**). (**G**) Representative ICC images of iCMs generated using polycistronic MGT constructs plus Hand2; (**H**) Statistical analysis of the ICC data in G. Scale bar, 200 μm. For each experiment, n = 3–4; averaged numbers from technical triplicates, or at least 10 random ICC fields were used for statistics. All data are mean ± SEM. Statistical analysis was performed with one-way ANOVA followed by Tukey’s test. * *p* < 0.05, ** *p* < 0.01, *** *p* < 0.001.

Initial iCM reprogramming cocktail included either separate M/G/T [6,7] (using Mi2) or M/G/T plus Hand2 (using Mi4) [5], and led to varied reprogramming efficiency between research groups. We, thus, studied the role of Hand2 in iCM reprogramming from MEF, using different Mef2C isoforms. We first evaluated the reprogramming efficiency using separate Mi2/G/T or Mi4/G/T in the presence or absence of Hand2. Our flow cytometry analysis indicated that Mi2/G/T or Mi4/G/T factors generated similar percentage of αMHC-GFP+ and cTnT+ cells. Addition of Hand2 resulted in a significant decrease of αMHC-GFP+ cells, while dramatically increased the percentage of cTnT+ cells, after induction of both separate factor cocktails (Figure 4A,B). Interestingly, the percentage of αMHC-GFP and cTnT double positive cells, remained similar between Mi2GT and Mi4GT groups cocktails (Figure 4A,B). Consistent with flow analysis, fluorescent staining experiments showed a reduced αMHC-GFP percentage in both Mi2/G/T/H and Mi4/G/T/H groups, compared to the Mi2/G/T and Mi4/G/T groups, respectively. In contrast, the percentage of cTnT+ cells increased in the Mi2/G/T/H group as compared to the Mi2/G/T group (Figure 4C,D). These results suggested that when using the M/G/T separate vectors, addition of Hand2 increased the efficiency of both isoforms of Mef2C mediated iCM reprogramming.

### 3.5. Hand2 Enhanced Mef2C Isoform 2 but Not Isoform 4 Mediated iCM Reprogramming from MEF When Using Polycistronic Constructs

We next explored the role of Hand2, together with the Mi2GT and Mi4GT polycistronic constructs for generation of iCMs. Consistent with previous results, percentage of αMHC-GFP+ and cTnT+ iCMs was higher in the Mi2GT treated group than that in the Mi4GT group (Figure 4F and Figure 2D,E). Addition of Hand2 increased cTnT%, in combination with Mi2GT but not with Mi4GT. Delivery of Mi2GT plus Hand2 induced a higher percentage of both αMHC-GFP+ and cTnT+ cells than that from Mi4GT plus Hand2 treatment (Figure 4E,F). ICC experiments showed similar results that a Mi2GT plus Hand2 treatment induced the highest reprogramming efficiency, among all other groups (Figure 4G,H). Taken together, our results showed that the delivery of Mef2C isoform2 in a polycistronic construct can further enhance the efficiency of direct iCM reprogramming with the addition of Hand2.

## 4. Discussion

In this study, we identified two Mef2C isoforms that were highly enriched in the murine heart and compared the effects of these two isoforms on cardiac reprogramming, using various origins of fibroblasts. We discovered that the reprogramming efficiency was relatively higher when the Mef2C isoform 2 was used and delivered to MEFs in polycistronic construct. In comparison, forced expression of Mef2C isoform 2 and 4 together with Gata4 and Tbx5 achieved by separate constructs lead to similar reprogramming efficiency. Addition of Hand2 further enhanced MGT induced iCM conversion, when using Mef2C isoform 2 in polycistronic construct.

Since the first report [7] that exogenous expression of the three cardiac transcription factors Gata4, Mef2C, and Tbx5 can directly convert mouse fibroblasts into functional iCMs without going through a PSC-like state, much effort has been taken to further improve the reprogramming efficiency. We and others have studied the cellular and molecular mechanisms underlying this cell fate conversion and have demonstrated that a battery of manipulations, such as modification of TFs and their structures, miRNAs, small molecules, and epigenetic factors, further enhance the iCM conversion [9,13,14,15,16,17,18,19,20,30,31]. The advances in cardiac reprogramming offer a great potential for generating high quality and quantity CMs and is, thus, promising for heart regeneration and disease modeling. However, due to differences, including laboratory protocols, reprogramming factor combinations, ways of virus production and delivery, starting fibroblast origins and methods to evaluate the reprogramming process, the iCM conversion ratio varied among the research groups. In this study, we found that two main Mef2C isoforms were used by research groups, which was one of the reasons for such reprogramming variation. Our results demonstrated that the Mef2c variant with α2 and γ splicing exon, coding for Mef2C isoform 2, gave rise to improved reprogramming efficiency in polycistronic construct transduced MEFs.

We also found that the different origins of fibroblasts reacted differently to the two Mef2C isoforms during reprogramming. The Mi2-mediated enhancement of iCM reprogramming was only discovered in MEFs but not CFs and TTFs. Intriguingly, this enhancement in MEFs can only be found when using polycistronic MGT rather than separate M/G/T (Figure 2C and Figure 4B), further indicating the importance of reprogramming factor stoichiometry, as previously reported [9]. Additionally, cellular plasticity and heterogeneity of MEFs might also contribute to such isoform-specific effect of iCM generation from MEFs. Our results also suggested the importance of the reprogramming factors and starting the fibroblast before designing reprogramming experiment. To achieve higher reprogramming efficiency, Mi2GT would be a better choice if MEFs is used.

In the developing heart, Hand2 is expressed at the same developmental stage as M, G, and T [32] and physically interacts with M, G, T to activate the cardiac gene expression [33,34,35]. The exact role of Hand2 to the MGT-mediated iCM reprogramming remains less studied. Hand2 has been reported to promote cardiac reprogramming and enhances the generation of iCMs in vitro [5,36]. Furthermore, consistent with this observation we discovered that Hand2 exerted this role when separate M/G/T factors or only Mi2 in polycistronic constructs were used (Figure 4). Of note, we discovered that when using separate factors, the Hand2 retrovirus can be directly added to the cultured cells, together with retrovirus encoding for other reprogramming factors. In comparison, Hand2 retrovirus could be added 24 h after delivery of retrovirus harboring polycistronic constructs, to achieve optimal reprogramming efficiency. This might be because the cultured cells will preferentially uptake the retrovirus harboring the small sizes of the construct.

In summary, we studied the role of different Mef2C isoforms for generating iCMs in different experimental contexts, including single factor construct versus polycistronic construct, different starting fibroblasts and reprogramming factor cocktails. We identified that the Mef2C isoform 2 is superior in the polycistronic construct, to convert MEFs to iCMs, together with Hand2. For fCFs, ExCFs, and ExTTFs, the two Mef2C isoforms yield similar reprogramming efficiency using polycistronic constructs. Therefore, our research clarifies some discrepancies in the iCM field, and can help with the choice of reprogramming platforms for basic and translational studies.
